# *Echinococcus multilocularis* in Kyrgyzstan: similarity in the Asian EmsB genotypic profiles from village populations of Eastern mole voles (*Ellobius tancrei*) and dogs in the Alay valley

**DOI:** 10.1017/S0022149X15000474

**Published:** 2015-07-03

**Authors:** E. Afonso, J. Knapp, N. Tête, G. Umhang, D. Rieffel, F. van Kesteren, I. Ziadinov, P.S. Craig, P.R. Torgerson, P. Giraudoux

**Affiliations:** 1 Chrono-environnement Laboratory, UMR 6249 CNRS, University of Franche-Comté, 16 route de Gray, F-25030Besançon, France; 2 ANSES, Nancy Laboratory for Rabies and Wildlife, National, Wildlife Surveillance and Eco-epidemiology Unit, Technopôle Agricole et Vétérinaire, B.P. 40009, 54220Malzéville, France; 3 Cestode Zoonoses Research Group, School of Environment and Life Sciences, University of Salford, M5 4WT Salford, UK; 4 Section of Epidemiology, Vetsuisse Faculty, University of Zurich, Switzerland; 5 Institut Universitaire de France, Paris, France

## Abstract

*Echinococcus multilocularis* is a cestode that causes human alveolar echinococcosis, a lethal zoonosis of public health concern in central Asia and western China. In the present study, one of 42 Eastern mole voles (*Ellobius tancrei)* caught in Sary Mogol (Alay valley, southern Kyrgyzstan) presented liver lesions with *E. multilocularis* from which the EmsB target was amplified. The Asian profile obtained was almost identical to one amplified from domestic dog faeces collected in a nearby village. This observation adds additional information to the potential role of *E. tancrei* in the transmission of *E. multilocularis,* and to the known distribution range of *E. multilocularis* (Asian strain) in central Asia.

## Introduction

The taeniid cestode *Echinococcus multilocularis* is the causative agent of human alveolar echinococcosis (AE), a potentially lethal helminthic zoonosis (Eckert & Deplazes, 2004). Although AE is a rare disease within the distribution range of the parasite, several endemic areas have been reported in North America, Europe and Asia (Vuitton *et al.*, 2003). *Echinococcus multilocularis* has a complex life cycle that involves carnivores (principally foxes) as definitive hosts, and cricetid rodents (e.g. *Microtus* spp.) or lagomorphs (e.g. *Ochotona* spp.) as intermediate hosts. Dogs are also good definitive hosts. The assemblage of wildlife host communities varies according to ecological features on multiple spatial scales (Giraudoux *et al.*, 2006). From a genetic point of view, *E. multilocularis* appears as an organism with low polymorphism (Haag *et al.*, 1997; Eckert *et al.*, 2001). However, distinct European, Asian and North American genotypes have been described (Bretagne *et al.*, 1996; Bart *et al.*, 2006) and the geographical location of the transitional zone between Asian and European genotypes, somewhere between eastern Europe and western China, is currently unknown. Furthermore, a tandemly repeated microsatellite, EmsB, has been used to describe the relative diversity of parasite genetic profiles on both regional and local scales (Knapp *et al.*, 2007, 2008, 2009).

Kyrgyzstan is one of the five republics of central Asia that, with northern Iran, eastern Turkey and Caucasia, provides the geographical link between the transmission foci of Asia and continental Europe. However, nothing is known about the genotypes of *E. multilocularis* circulating in the area, which theoretically may belong either to the Asian or the European clades, or both. In Kyrgyzstan, cystic echinococcosis caused by *E. granulosus*, is a national public health concern across the whole country (Torgerson *et al.*, 2006). The highest incidences of human alveolar echinococcosis, however, are currently recorded in the sub-national administrative regions of Issyk-kul, Naryn and Osh, the latter including the Alay valley (Usubalieva *et al.*, 2013). In the Alay valley (altitude 2900–3500 m) land cover is mostly Alpine grassland. *Echinococcus multilocularis* definitive hosts are the red fox (*Vulpes vulpes)* and domestic dogs (Ziadinov *et al.*, 2008, 2010). In terms of potential prey biomass, the three dominant species in local small mammal assemblages are: *Microtus gregalis* (the narrow-headed vole), *Cricetulus migratorius* (the grey dwarf hamster) and *Ellobius tancrei*, (the Eastern mole vole) (Giraudoux *et al.*, 2013 and unpublished). Although, historically, *M. gregalis* and *E. tancrei* have been found to be infected naturally in Kyrgyzstan (Gagarin *et al.*, 1957; Tokobaev, 1959), their relative contribution to *E. multilocularis* transmission is still unknown. *Ellobius tancrei* has a wide distribution range, stretching from north-eastern Turkmenistan and eastern Uzbekistan through China and Mongolia (Batsaikhan & Tinnin, 2008). More than 50 years ago this species was already recorded as being infected naturally with *E. multilocularis* in Kyrgyzstan (Tokobaev, 1959), but in the original paper it was likely confused with *E. talpinus*, the Northern mole vole, which actually is not present in Kyrgyztan. No other mention since then of *E. tancrei* voles infected by *E. multilocularis* could be found in the literature. However, population surges of this species have been observed regularly, for instance in the Alay valley, the Tien Shan (Narati area, Xinjiang, China) and the Altai Mountains (Giraudoux *et al.*, 2008, 2013 and unpublished).

**Fig. 1:**
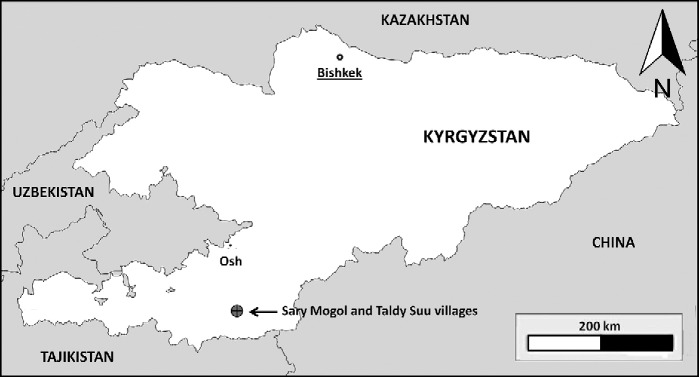
Map of Kyrgyzstan to show the study site (circled) in the Alay valley.

Here we report infection of *E. tancrei* in Sary Mogol village (39°40′33.06″N, 72°53′02.06″E) (fig. 1). Furthermore, dog faeces were sampled and tested for *E. multilocularis* in the same area, and one of them was used to compare genetic profiles. Those genotypic profiles were then compared to other *E. multilocularis* isolates from Eurasia and North America.

## Materials and methods

**Fig. 2:**
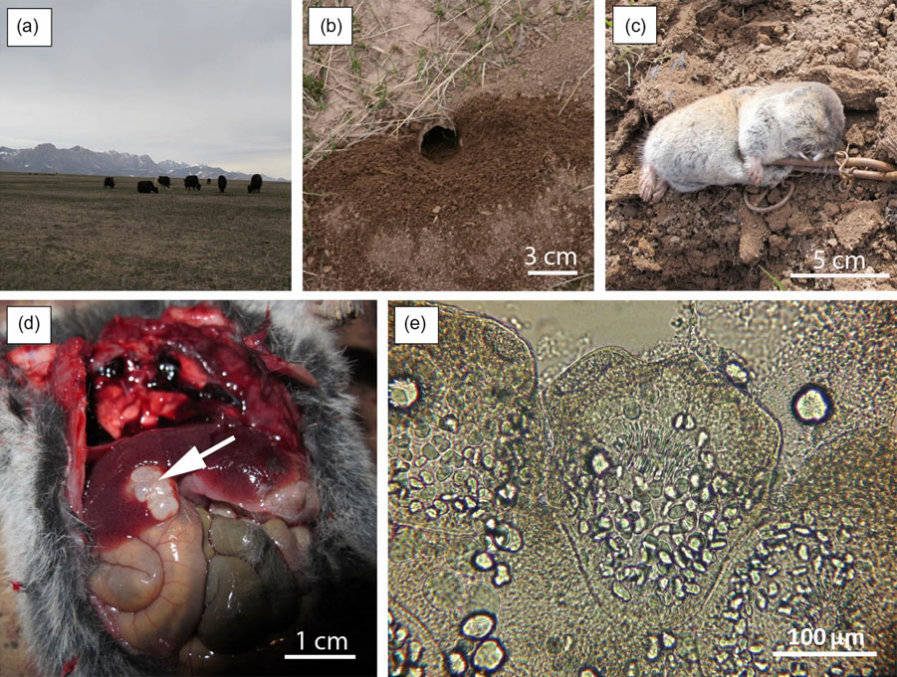
(a) The landscape of the Alay valley with (b) a burrow entrance of *Ellobius tancrei*; (c) an entire specimen of *E. tancrei* caught in a tong trap; (d) liver lesion (arrowed) caused by *Echinococcus multilocularis*; (e) invaginated protoscolex of *E. multilocularis*.

In May 2012, a total of 42 *Ellobius* specimens were trapped within the periphery of Sary Mogol village using tong traps, in an area of about 0.53 ha (72°53′27.78″E, 39°40′50.952″N) at an altitude of 3000 m. As in every other household of this area, the hamlet was surrounded by Alpine grassland and farmland (fig. 2a). Eastern mole voles were identified to the specific level using conspicuous and typical morphometric criteria (short and soft fur, small eyes, long and straight incisors extending far forward of the nasal cavities; fig. 2c). All animals were weighed, measured and sexed in a field laboratory. Rodent eyeballs were collected to assess their relative age by using their dry crystalline weight, and were preserved in 5% formalin (Kozakiewicz, 1976). At necropsy, the liver and lungs were examined macroscopically for any lesions. When lesions were found, samples were collected and stored in a 90% alcohol solution. The presence of protoscoleces was assessed under microscopy after a puncture into the lesion with a syringe. Rodent carcases were preserved in 10% formalin for reference collection.

Dog faeces were sampled in Sary Mogol and other villages over the same period. *Echinococcus multilocularis* DNA was amplified from dog faeces found in Taldy Suu village (72°58′15.75″E, 39°42′24.41″N) situated 7.4 km from the small mammal sampling spot (see van Kesteren *et al.*, 2013).

Total genomic DNA from the rodent liver lesion was extracted by using the High Pure PCR Template Preparation kit (Roche Diagnostics, Mannheim, Germany), as recommended by the manufacturer. The *Echinococcus* species determination was done with DNA amplification by polymerase chain reaction (PCR) and sequencing of the mitochondrial DNA (mtDNA) fragment of the *nd1* gene (primers ND1_Fwd: 5′-AGATTCGTAAGGGGCCTAATA-3′ and ND1_Rev: 5′-ACCACTAACTAATTCACTTTC-3′; Bowles & McManus, 1993) and compared to the GenBank database. Sequencing using the Sanger method was performed from the two ND1 primers, in order to obtain a consensus sequence. For the dog faecal sample, DNA was extracted using a Qiagen stool mini kit (Qiagen, Hilden, Germany) following the manufacturer's instructions but using 1 g of faeces. The positive dog faecal sample from Taldy Suu was also amplified for the *nd1* gene.

Genotyping of parasite samples was performed by amplification of the tandemly repeated microsatellite EmsB as described previously (Knapp *et al.*, 2007) and modified (Umhang *et al.*, 2014). Briefly, the reaction was performed in a 25 μl reaction mixture, containing 200 μm of each deoxynucleoside triphosphate (dNTP), 0.4 μm fluorescent forward primer EmsB A (5′FAM-GTGTGGATGAGTGTGCCATC-3’), 0.7 μm classical reverse primer EmsB C (5′-CCACCTTCCCTACTGCAATC-3′) and 0.5 U of Platinum *Taq* DNA polymerase enzyme (Life Technologies, Foster City, California, USA), with the addition of Platinum 1 ×  PCR buffer (Life Technologies). The amplification reaction was performed in a Veriti thermocycler (Life Technologies), under the following conditions: a pre-amplification step of 94°C for 2 min; followed by 45 cycles with a denaturing step at 94°C for 30 s, annealing at 60°C for 30 s and extension at 72°C for 1 min; with a final elongation at 72°C for 45 min. The PCR products were analysed by fragment size analysis using an ABI Prism 310 apparatus and the GeneMapper 4.1 software (Life Technologies, Carlsbad, California, USA). The Kyrgyz sample isolated from *E. tancrei* was compared to a database composed of 1084 genotyped samples from Europe (France, *n*= 537; Germany, *n*= 88; Switzerland, *n*= 109; Austria, *n*= 99; Slovakia, *n*= 63; Czech Republic, *n*= 66; and Poland, *n*= 94), from Asia (Tibetan plateau in China, *n*= 5; Hokkaido in Japan, *n*= 6) and from North America (Canada, *n*= 1; Alaska, *n*= 13). The Kyrgyz positive dog faecal sample contaminated by *E. multilocularis* (*n*= 1) was included, and a sample of *E. granulosus* sensu stricto as an outgroup (*n*= 2). The genetic distance amongst samples was assessed by Euclidean distance between EmsB profiles. As described previously, two samples were considered as identical when the genetic distance was below 0.08 (Knapp *et al.*, 2007).

## Results

**Fig. 3:**
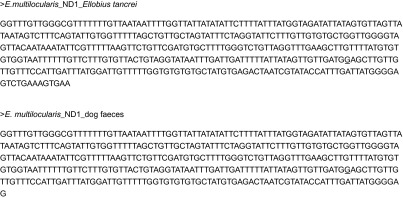
Part of the *nd1* gene sequenced from the *Ellobius tancrei* liver lesion and from the positive dog faecal sample contaminated by *Echinococcus multilocularis*. The underlined nucleotide corresponds to the mutation position in comparison to the AB018440.2 complete mitochondrial genome referenced.

**Fig. 4:**
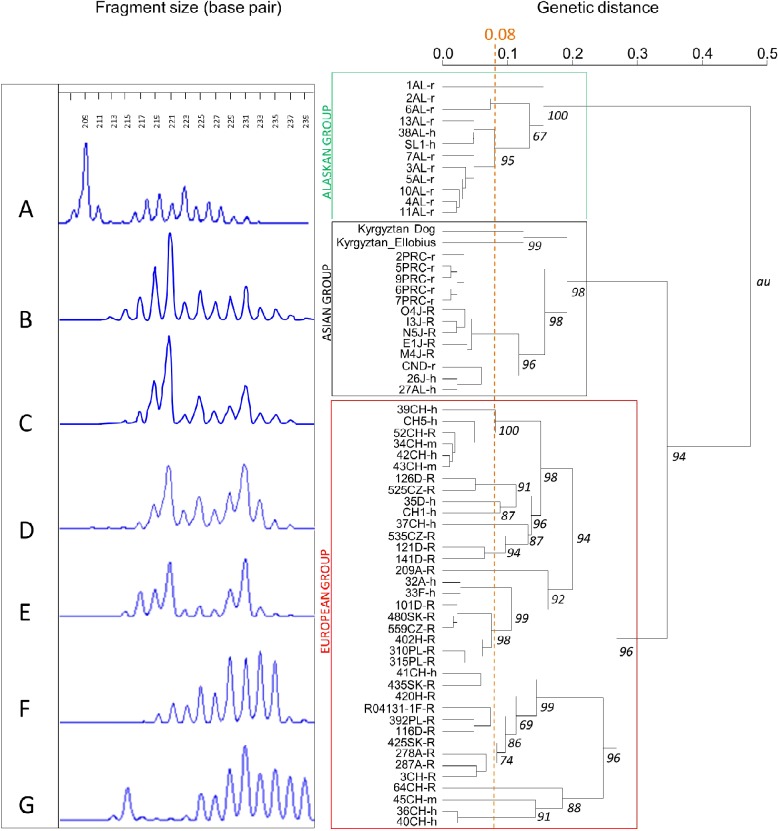
EmsB profiles of *Echinococcus multilocularis* from samples in (A) Alaska; (B) dog faeces, Kyrgyzstan; (C) liver of *Ellobius tancrei*, Kyrgyztan; (D) liver of *Microtus limnophilus*, Siqhu, Tibetan plateau, Sichuan, China; (E) fox intestine, Hokkaido, Japan; and (F, G) fox intestine, Europe. The dendrogram represents the similarities between samples, with bootstrap values (B = 1000) at each node and the limit of high similarity being 0.08 (Knapp *et al.*, 2007).

Among the 42 individuals, 15 were females and 27 males. The body weight ranged from 47 to 77 g and crystalline dry mass from 0.45 to 3.8 mg. One *Ellobius* specimen, an adult male, was caught by hand and brought by children from the hamlet. Its body weight was 62 g and crystalline dry mass 1.1 mg. This specimen was the only individual that presented larval cysts of *E. multilocularis*. It showed two liver lesions (12–18 mm in diameter; fig. 2d). Protoscoleces were found after examining cyst vesicle fluid under a light microscope (fig. 2e). For both the *Ellobius* specimen and the dog faecal sample, the amplification of the mtDNA fragment of the *nd1* gene allowed us to generate a 400-bp consensus sequence. The two isolates had 100% identity with each other and presented 99% identity with the *nd1* sequence from the complete mitochondrial genome (AB018440.2). One mutation was observed (position 8012 G/A mutation) in the referenced sequence in both the forward and reverse sequences, in comparison to the other sequences referenced in the GenBank database for the *E. tancrei* sample and the dog faeces extract (see sequences in fig. 3). This mutation was observed amongst, for example, a Polish sample (GenBank reference: AJ132908.1) and Chinese samples (Xinjiang sample: EU704124.1 and Sichuan: EU704123.1), these reference samples having the nucleotide A at the position 8012 in the *nd1* gene, and the Kyrgyz samples a nucleotide G. The presence of the mutation was confirmed by performing the sequencing twice. In comparison to the EmsB database (*n*= 1084 samples) no identical samples ( < 0.08 of genetic distance) were clustered with the Kyrgyz sequences (from *E. tancrei* and the dog faecal samples), but the two Kyrgyz sequences were clustered together with a genetic distance of 0.12. They can subsequently been considered as similar strains but not identical, perhaps due to poor DNA quality (fig. 4). Moreover, the two samples were linked with Tibetan (China) and Hokkaido (Japan) samples, and one Alaskan sample, with a genetic distance ranging from 0.17 to 0.24 (fig. 4), but with neither the European nor American isolates.

## Discussion

The current results add further information about the natural infection of the Eastern vole mole, *E. tancrei*, with *E. multilocularis*, first discovered more than 50 years ago. These findings based on EmsB genotyping indicate, first, that the two isolates (vole and dog) found in our study belong to the Asian strain of *E. multilocularis*, hence extending the western limit of the known distribution range of this genotype in central Asia. The Pamir Mountain range is situated in altitudinal continuity with the Tibetan plateau but, due to its complex high-altitude ranges, might have been considered a biogeographical barrier to the spread of the eastern Asian strain of *E. multilocularis* to the central Asian republics – a hypothesis that is refuted here. Second, very similar strains were found in dog faeces and the *E. tancrei* specimen in the study area, and the common mutation first described in the present study emphasized, as a fingerprint, the involvement of *E. tancrei* and dogs in the local parasite cycle. The occurrence of this mutation amongst Asian *E. multilocularis* isolates needs further studies to be understood. Associated with the fact that *E. tancrei* could be trapped at less than 10 m from house walls, and all of them at less than 100 m, this indicates that a synanthropic cycle involving dogs and the Eastern mole vole may exist, not excluding the contribution of other small mammal potential host species (e.g. *M. gregalis, C. migratorius*) that were also observed not only in habitats remote from villages but also in the close vicinity of houses, where *Mus musculus* was also captured. Large population densities of both dogs and *E. tancrei* were observed in the Alay valley. *Ellobius tancrei* abundance has been shown to increase with grassland vegetation biomass (Giraudoux *et al.*, 2013). This leads to the maintenance of larger vole populations in farmland that surrounds villages, where barley is grown, and in hay fields close to villages, with vole population spillover into villages. Moreover, 38–74% of households have at least one dog in the villages studied in the Alay Valley (van Kesteren *et al.*, 2013), which leads to a high concentration of potentially infective dog faeces. This should be added to a large red fox population in the area, with tens of fox dens found at less than 1–2 km from villages (Giraudoux and Rieffel, pers. obs.), which may also feed the sustainable transmission of *E. multilocularis* (however, see Liccioli *et al.*, 2015). Third, the only specimen of *E. tancrei* found to be infected by *E. multilocularis* was also the only specimen caught by hand by children. This might indicate that the animal found infected in the present study might have been caught not by chance but as the result of an increased vulnerability to capture induced by the parasite. This possibly altered host-behavioural aspect of the transmission ecology of *E. multilocularis* appears not to have been mentioned previously in the literature, and should be investigated carefully, using appropriate methods.

## Acknowledgements

We thank Alexander Mastin, Mike Rogan and Bermet Mytynova for their help in obtaining the domestic dog sample.

## Financial support

Financial support was received from the Wellcome Trust (#094325/Z/10/Z programme). This research has been conducted within the context of the GDRI (International research network) ‘Ecosystem health and environmental disease ecology’.

## Conflict of interest

None.
